# Virulence and antibiotic-resistance genes in *Enterococcus faecalis* associated with streptococcosis disease in fish

**DOI:** 10.1038/s41598-022-25968-8

**Published:** 2023-01-27

**Authors:** Tasmina Akter, Md. Najmul Haque, Rakib Ehsan, Sulav Indra Paul, Md. Javed Foysal, Alfred Chin Yen Tay, Md. Tofazzal Islam, Md. Mahbubur Rahman

**Affiliations:** 1 Institute of Biotechnology and Genetic Engineering, Bangabandhu Sheikh Mujibur Rahman Agricultural University, Gazipur, 1706 Bangladesh; 2 Department of Fisheries Management, Bangabandhu Sheikh Mujibur Rahman Agricultural University, Gazipur, 1706 Bangladesh; 3 Department of Animal Science and Veterinary Medicine, Bangabandhu Sheikh Mujibur Rahman Science and Technology University, Gopalganj, 8100 Bangladesh; 4 School of Molecular and Life Sciences, Curtin University, Bentley, WA Australia; 5 Department of Genetic Engineering and Biotechnology, Shahjalal University of Science and Technology, Sylhet, 3114 Bangladesh; 6 Marshall Centre for Infectious Disease Research and Training. School of Biomedical Sciences, University of Western Australia, Perth, WA Australia

**Keywords:** Microbiology, Molecular biology, Diseases

## Abstract

*Enterococcus faecalis* is associated with streptococcosis like infection in fish. A whole-genome sequence study was conducted to investigate the virulence factor and antibiotic-resistance genes in three fish pathogenic *E. faecalis*. Genomic DNA was extracted from three strains of *E. faecalis* isolated from streptococcosis infected Nile tilapia (strains BF1B1 and BFFF11) and Thai sarpunti (strain BFPS6). The whole genome sequences of these three strains were performed using a MiSeq sequencer (Illumina, Inc.). All three strains conserved 69 virulence factor such as genes associated with protection against oxidative stress, bacterial cell wall synthesis, gelatinase toxin, multiple biofilm-associated genes and capsule producing genes. Moreover, 39 antibiotic-resistance genes against sixteen major groups of antibiotics were identified in the genome sequences of all three strains. The most commonly used antibiotic Tetracycline resistance genes were found only in BFPS6 strain, whereas, Bacteriocin synthesis genes were identified in both BFFF11 and BFPS6 strain. Phylogenetic analysis revealed that strains BF1B1 and BFFF1 form a different cluster than BFPS6. This is one of the first whole-genome sequence study of fish pathogenic *E. faecalis*, unfold new information on the virulence factor and Antibiotic resistance genes linked to pathogenicity in fish.

## Introduction

Aquaculture is one of the fastest-growing sub-unit of the animal food sector that plays a crucial role in global food security^1^. Global fish production has increased three folds during the last two decades from 34 Mt in 1997 to 112 Mt in 2017 and reached 179 Mt in 2018 with the upward trend expected to continue in the near future^2,3^. Recent technological advancement and intensification have expedited the global boom of aquaculture^1^. However, disease is considered one of the foremost threats in intensive aquaculture^4^. Streptococcosis, columnaris, vibriosis, hemorrhagic septicemia, edwardsiellosis etc. are the major bacterial diseases that most frequently occurs in tilapia and other aquaculture species^5–7^. Among these, streptococcosis is one of the vital diseases in tilapia caused by a complex group of bacteria including *Streptococcus iniae, S. agalactiae*, *Lactococcus garviae*, *Vagococcus salmoninarum* and *Enterococcus* spp.^5,8,9^. The first pathogenic *Enterococcus* sp. has been identified from streptococcosis like infection in yellowtail (*Seriola quinqueradiata*) which was named *Enterococcus seriolicida*^10^. Later *E. seriolicida* was reclassified as *Lactococcus garvieae*. Morita, et al.^11^ detected *L. garvieae* as the causal agent of septicemic fatal hemorrhagic disease in fish. Recently, pathogenic *Enterococcus faecalis* were diagnosed from streptococcosis like infection in Nile tilapia in Egypt, India and Bangladesh^12–14^ and in silver barb (*Barbonymus gonionotus*) in Bangladesh^8^. Like *Streptococcus, E. faecalis* is a Gram-positive, cocci belong to the family Enterococcaceae that causes disease in animals, and humans. Our previous studies based on 16S rRNA were conducted to identify few randomly selected strains of *E. faecalis* such as BFFF11, BFF1B1, BFTS15, BFTS17, BFTS22, BFTS23, BFTS25, BFTS27 and BFTS29 isolated from streptococcosis infected Nile tilapia in Bangladesh. *E. faecalis* (i.e. strains BFPS1, BFPS3, BFPS6 and BFPS13) were also identified from streptococcosis infected silver barb (*B. gonionotus*) fish^8^. Among the strains mentioned earlier, BFFF11, BFF1B1 and BFPS6 appears to be more pathogenic, hence, these three strains were focused in the current study. Most of the previous studies used *16S rRNA* gene sequencing to identify fish pathogenic *E. faecalis*, However, very few studies on the same context were performed based on the whole-genome sequence.

The virulence gene of a pathogen determines the pathogenicity to beat the host defense system and establish disease^15^. The antimicrobials resistance is another system that plays a crucial role in the virulence mechanism of a pathogen^15^. The development of antibiotic-resistance genes enables the pathogenic bacteria to adapt and survive in a new environment, particularly during antibiotic treatment^15^. The increased use of antibiotics in commercial aquaculture enforces the pathogenic bacteria to possess antimicrobial resistance genes^16^. Although several virulence factors and multidrug resistance genes were reported from human and animal pathogenic *E. faecalis*^17,18^, no genes associated with virulence activity and antimicrobial resistance were identified from this fish pathogen. Accurate detection of organism, it’s pathogenicity and antimicrobial resistance would be crucial to take timely preventive measures so that disease outbreak can be halted. The genome announcement of the strains BFFF11 and BFPS6 were performed in our previous studies^8,19^, where details on the virulence and antibiotic resistance genes were not analyzed. We hypothesized that whole-genome sequence as one of the latest biotechnological tools, would be able to detect the virulence factors of the pathogenic *E. faecalis*. Therefore, the objectives of this study were to identify the virulence factors and antibiotic-resistance genes profile through whole-genome sequencing of the fish pathogenic *E. faecalis* strains isolated from diseased fishes in Bangladesh.

## Results

### Isolation, phenotypic identification, pathogenicity and antibiogram profiling

*Enterococcus faecalis* strains BFF1B1, BFFF11 and BFPS6 were cultured in Streptococcus selective agar media (Himedia, India). The culture characteristics such as colony, morphological, physiological and biochemical characteristics of these strains BFF1B1, BFFF11 and BFPS6 were summarized in the Supplementary Table S1. All of these strains were Gram-positive, cocci shaped, non-motile and produce dark red colonies while culturing in agar media. They were catalase, oxidase, urease, Arabinose, Fructose, Inositol, Inulin, Raffinose and Xylose negative. They produced β hemolysis in sheep blood agar media. The antibiogram profiling of these strains were performed using disk diffusion methods against eleven commercial antibiotics (Supplementary Table S2). We observed that all strains were resistant to Amoxicillin, Ampicillin, Cefuroxime, Erythromycin and Penicillin. Moreover, the strain BFPS6 was also resistant to Cefradine. Furthermore, *in-vivo* challenge test revealed that BFFF11, BFF1B1 and BFPS6 were identified as highly virulent (≥ 80% mortality) strains (Supplementary Table S1).

### General features of the genome sequences

The genomic characteristics of the three strains of *E. faecalis* associated with streptococcosis in fish were studied using a subsystem set of seed viewer. The genome sizes of the strains BFF1B1, BFFF11 and BFPS6 were 2.76, 3.07 and 2.87 Mb, respectively. The GC content (%) of the strains was substantially the same (Table 1). The predicted coding sequences (CDSs) by RAST in the strains BFF1B1, BFFF11 and BFPS6 were 2588, 2870 and 2743, respectively. Only 30–52% CDSs in each strain can be functionally categorized into 250–357 subsystems. On the other hand, PATRIC analysis revealed 2631, 2949 and 2745 CDS in the strains BFF1B1, BFFF11 and BFPS6, respectively. In the subsystems, carbohydrates, amino acids and derivatives and protein metabolism had a higher number of functional genes in all of the studied bacteria. None of the strains carried genes related to photosynthesis and secondary metabolism. An overview of the genome features of the *E. faecalis* strains and their subsystem statistics were shown in Table 1 and Supplementary Fig. S1.

**Table 1:** General genomic features of the *E. faecalis* strains BFFF11, BFF1B1 and BFPS6 obtained from RAST and PATRIC analysis.

Parameters	Subsystem statistics		
Genome feature	BFF1B1	BFFF11	BFPS6
Size (bp)	2,761,629	3,067,042	2,868,292
GC content (%)	37.6	37.4	37.5
N50	384,233	343,888	270,331
L50	2	4	2
No. of coding sequences (CDs)	2588	2870	2743
No. of RNA	63	66	60
No. of subsystems	353	357	250
Fine Consistency	97.3	97.4	97.3
CDS	2631	2949	2745
Repeat region	87	111	104
tRNA	51	55	56
rRNA	9	6	5
**Protein feature**			
Proteins with functional assignments^1^	2131	2283	2206
Hypothetical proteins^1^	500	666	539
Proteins with GO assignments	540	581	558
Proteins with genus-specific family assignments^2^	2556	2777	2688
Proteins with cross-genus family (PGfam) assignments^2^	2582	2822	2716
**Speciality genes**			
Virulence factor	33	36	34
Transporter	33	54	43
Drug target	11	11	11
Antibiotic resistance	32	34	34

^1^values indicate with KEGG annotation.

^2^Proteins with PATRIC genus-specific family (PLfam) and cross-genus-specific family (PGfam) assignments (Brettin et al*.*^47^).

**Figure 1:**
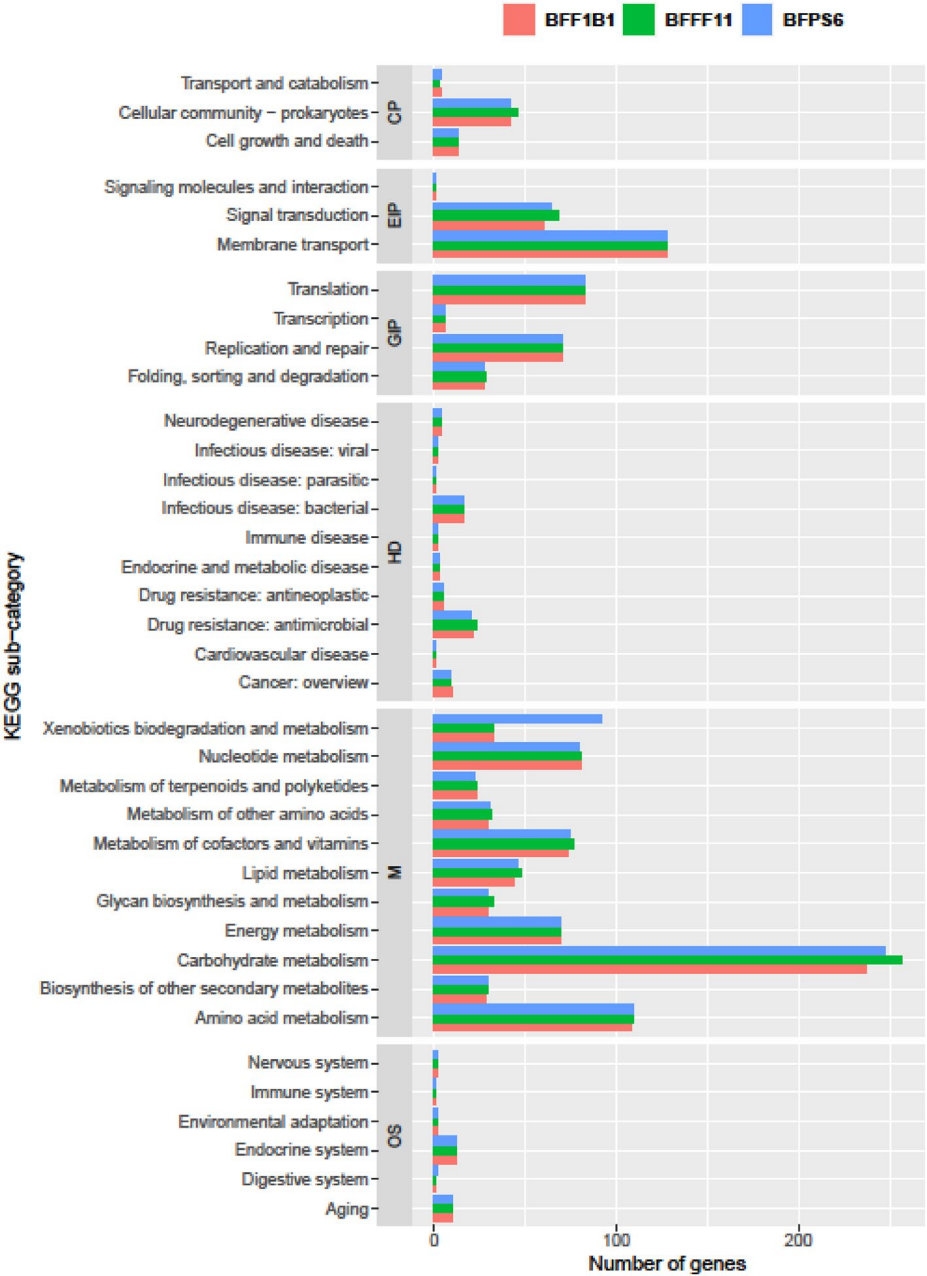
The number of genes categorized by KEGG functional annotation of *E. faecalis* strains BFF1B1, BFFF11 and BFPS6.

According to the Kyoto Encyclopedia of Genes (KEGG) study, the CDSs were classified into six sub-categories including cellular processes (CP), environmental information processing (EIP), genetic information processing (GIP), human disease (HD), organisms system (OS), and metabolism (M) (Fig. 1). The strains BFF1B1, BFFF11 and BFPS6 were found to conserve 1280, 1325 and 1356 CDSs, respectively, that were further divided into 37 functional KEGG sub-categories according to the aforementioned six categories (Fig. 1 and Supplementary Table S3). Notably, all of the strains contained a significantly higher number of genes related to metabolic pathways (CDSs 753–826), followed by environmental information processing (CDSs 199–197) and genetic information processing (CDSs 186–187) (Supplementary Table S3).

**Figure 2:**
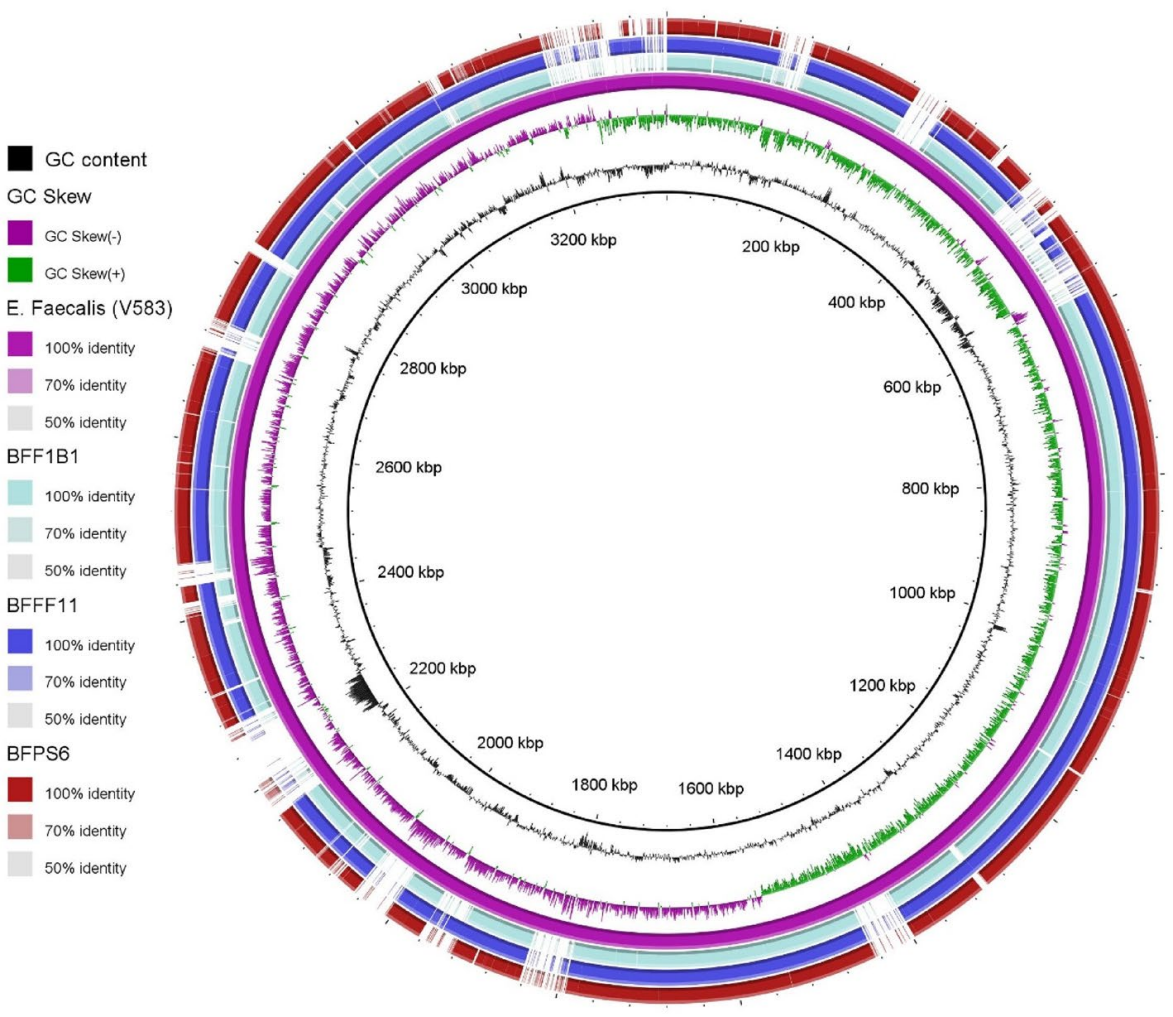
Blast atlas of three *Enterococcus faecalis* strain (BFFF1B1, BFFF1 and BFP6S6) mapped against reference sequence of *Enterococcus faecalis V583*. Blast atlas were generated by BRIG using both alignment length and identify cut off values minimum of 50%. The innermost two rings (first and second) represent GC content (black) whereas, the third ring shows GC skew (purble/ green). The remaining 4 rings (rings 4–7) represent a BLASTN comparison with complete genome of *E. faecalis* strains V583 (megenda ring which was used as reference genome) BFF1B1 (Deep sky), BFFF11 (Blue) and BFPS6 (Maroon).

A comparative genomic analysis was performed among the strains where *E. faecalis* V583 was used as a reference. The genomic map obtained from the BRIG comparison did not show large scale variation between the bacterial genome sequences, and a significant number of non-homologous regions were found around the reference genome with over 80% identity (Fig. 2). Most of these non-homologous regions might be linked to transposable elements.

### Virulence factor and biofilm formation-associated genes

**Figure 3:**
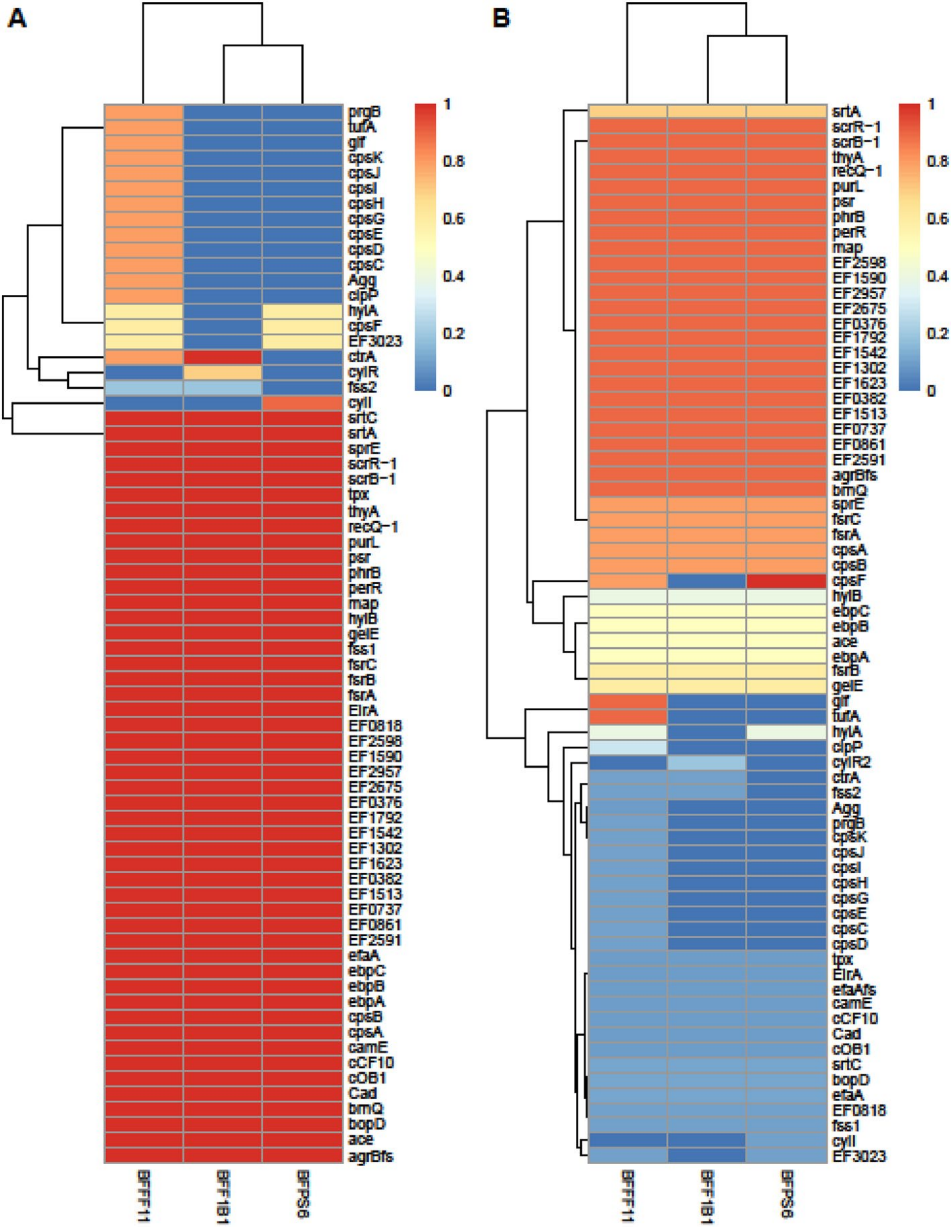
Heat maps of virulence genes. (**A**) Presence and absence of VG. Dark red = presence in all three strains, Light red = Presence in BFPS6, Orange = presence in BFFF11, Dark cream = presence in BFF1B1, Light cream = Presence in BFFF11 and BFPS6, Light blue = Presence in BFF1B1 and BFFF11 and Dark blue = absence of VG. (**B**) Identification of VG according to database. Dark red = Present in all database used for this study, Light red = PATRIC_Victors, Orange = VFDB and PATRIC_VFDB, Orange = VF and PATRIC_Victors, Dark cream = VF and PATRIC_VFDB, Light cream = VF and VFDB, PATRIC_VFDB, Light Blue = VFDB, Sky blue = VF, Dark blue = absence in database.

The degree of pathogenicity of microbes is greatly influenced by their virulence gene contents. In this study, a total of 69 virulence genes were identified in *E. faecalis* strains BFFF11, BFF1B1 and BFPS6 (Fig. 3a). Genes associated with protection against oxidative stress (*tpx, perR*), bacterial cell wall synthesis (*psr*) and gelatinase toxin (*gelE*) were identified in all of the three strains. Two extracellular hyaluronidases genes *hylA* and *hylB* that evade the phagocytosis process with macrophage persistence of host were identified only in the genome sequence of BFFF11, whereas *hylA* was found in both BFF1B1 and BFPS6 strains.

Many genes associated with biofilm formation were identified such as two aggregation substances (*agg* and *prgB*), endocarditis and biofilm-associated pili genes (*ebpA, ebpB, ebpC*), collagen adhesion precursor (*ace*), three proteolytic processing of a quorum-sensing system signal molecule precursors (*fsrA, fsrB* and *fsrC*), accessory regulator protein (*agrBfs*), sugar-sensing transcriptional regulator (*bopD*), serine protease (*sprE*) and two genes for sortase assembled pili (s*rtA* and *strC*). Although strain BFFF11 harbored all of the above biofilm-producing factors; two aggregation substance encoding genes *agg* and *prgB* were absent in the genomes of BFF1B1and BFPS6.

Numbers of virulence genes for DNA and protein synthesis were found in the genome sequences of all three strains, namely DNA repair enzymes synthesis gene (*recQ1* and *phrB*), purine metabolism gene (*purl*), thymidylate synthase (*thyA*), methionine aminopeptidase (*map*) and sucrose operon repressors genes (*scrB-1* and *scrR-1*). Among three strains, BFF1B1 and BFFF11 harbor *ctrA* gene that functions as a negative regulator of DNA replication.

Other virulence factors included sex pheromone associated genes (*cad, cCF10, camE*, *cOB1*), the cell wall adhesion expressed in serum gene (*efaA*), the enterococcal Rgg-like regulator gene, amino acid transport and synthesis regulators (*brnQ*), and gene associated with macrophage persistence (*ElrA*). Cytolysin toxin-producing genes *cylR2* and *cylI* was identified in BFF1B1 by VFDB and BFPS6 by PATRIC, however, these were not identified in the BFFF11 strain. Furthermore, 11 capsule producing genes (*cpsA* to *cpsK*) associated with anti-phagocytosis were identified in the strain BFFF11; among those only 3 genes (*cpsA*, *cpsB* and *cpsF*) were in the Thai sarpunti originated BFPS6 and only *cpsA* was found in the BFF1B1. Heat shock regulation protease gene (*clpP*), translation elongation factor (*tufA*) and UDP-galactopyranose mutase synthesis gene (*glf*) which play important roles in cell surface formation and infection cycle of pathogens were found only in the strain BFF1B1. A relatively large number of virulence genes were identified using the VFDB database, and the lowest was identified using the virulence finder (Fig. 3b).

### Antibiotic-resistance gene

A total of thirty-nine antibiotic-resistance genes belonging to sixteen different groups were identified among the strains of *E. faecalis* (Table 2). Except for four genes including *tet(M), tet(L), tet(S)* and *tet(45*), all of the genes were conserved by the genome sequences of all three strains of *E. faecalis.* Although tetracyclines, aminoglycosides, phenicol antibiotics resistant gene *YkkCD* and tetracyclines, glycylcyclines resistant gene *S10p* were conserved in all the genome sequences of present study strains, four genes such as *tet(M), tet(L), tet(S)* and *tet(45*) conferring resistance to tetracycline were identified only in the genome sequence of BFPS6 with 77.14–100% of identity.

**Table 2:** Acquired antibiotic resistance genes identified in the strains of E. *faecalis* obtained by ARG-ANNOT Nt, Resfinder and CARD.

Antibiotic resistance genes	BFFF11	BFF1B1	BFPS6	Antibiotic resistance group	Sources (NCBI accession No./Pubmed)
*efrA*^c, p^	+	+	+	Macrolide-lincosamide-streptogramin, rifamycin	CDO61513.1
*efrB*^p^	+	+	+	Macrolide-lincosamide-streptogramin, rifamycin	CDO61516.1
*YkkCD*^p^	+	+	+	Tetracyclines, aminoglycosides, phenicol	10,735,877
*LiaR*^p^	+	+	+	Daptomycin	AFK58562.1
*LiaS*^p^	+	+	+	Daptomycin	21,899,450;26,020,679
*LiaF*^p^	+	+	+	Daptomycin	21,899,450;26,020,679
*MprF*^p^	+	+	+	Daptomycin	19,289,517;16,723,576
*GdpD*^p^	+	+	+	Daptomycin	21,899,450
*PgsA*^p^	+	+	+	Daptomycin	22,238,576
*rpoC*^p^	+	+	+	Daptomycin	16,723,576
*rpoB*	+	+	+	Rifamycins	3,050,121
*rho*^p^	+	+	+	Bicyclomycins	8,466,900
*EF-G*^p^	+	+	+	Fusidic acid	17,980,694
*Alr*^p^	+	+	+	D-cycloserine	19,748,470;24,303,782
*Ddl*^p^	+	+	+	D-cycloserine	24,303,782;24,033,232
*EF-Tu*^p^	+	+	+	Elfamycins	364,475;9,678,602
*MurA*^p^	+	+	+	Fosfomycin	8,994,972
*folA, Dfr*^p^	+	+	+	Trimethoprim, Diaminopyrimidines	20,169,085;25,288,078
*Iso-tRNA*^p^	+	+	+	Mupirocin	7,929,087
*S12p*^p^	+	+	+	Streptomycin	7,934,937
*gidB*^p^	+	+	+	Streptomycin	17,238,915
*kasA*^p^	+	+	+	Isoniazid, triclosan	10,428,945
*S10p*^p^	+	+	+	Tetracyclines, glycylcyclines	26,124,155
*folP*^p^	+	+	+	Sulfonamides	15,673,783
*gyrA*^p^	+	+	+	Fluoroquinolones quinolones quinolines	9,293,187
*gyrB*^p^	+	+	+	Fluoroquinolones quinolones quinolines, aminocoumarin	21,693,461;22,279,180;9,293,187
*RlmA(II)*^p^	+	+	+	Macrolides, lincosamides	12,514,124;18,406,425
*FabK*^p^	+	+	+	Triclosan	10,910,344
*inhA, fabI*^p^	+	+	+	Isoniazid, ethionamide, triclosan	18,193,820;10,869,170;8,284,673
*VanG-type*^p^	+	+	+	Vancomycin	
*lsa(A)*^a, r, p^	+	+	+	Macrolide-lincosamide-streptogramin	AY225127
*mph(D)*^a, p^	+	+	+	Macrolide-lincosamide-streptogramin	NC_017312
*amp(S)*^a^	+	+	+	Beta-lactamases	NC_014932
*dfr(E)*^a, c, p^	+	+	+	Trimethoprim	NG_055770, AAD01867.1
*tet(M)*^a, r^	–	–	+	Tetracycline	DQ534550
*tet(L)*^a, r^	–	–	+	Tetracycline	FN435329
*tet(S)*^a^	–	–	+	Tetracycline	L09756
*tet(45)*^a, c^	–	–	+	Tetracycline	NG_048147

Here, a, r, c and p denote the gene identified by using the database ARG-ANNOT Nt, Resfinder, CARD and PATRIC, respectively. + and – indicate the gene presence and absence in the genome of the respective strains, respectively.

Macrolide-lincosamide-streptogramin (MLS) resistant genes *lsa(A)*, *RlmA(II)* and *mph(D)* were found in all of the *E. faecalis* strains. Similarly, two multidrug-resistant efflux pump conferring genes *efrA* and *efrB* were identified, found to be resistant against MLS and rifamycin antibiotics. Eight antibiotic-resistance genes included *LiaR, LiaS, LiaF, MprF, GdpD*, *PgsA, rpoB* and *rpoC* were found where seven of them were resistant against daptomycin and *rpoB* were resistant against rifamycin, daptomycin, rifabutin and rifampin drugs. All three strains harbored genes *kasA, FabK, inhA* and *fabI* were resistant to isoniazid and triclosan group of antibiotics. Furthermore, two genes *dfr(E)* and *folA* were identified, resistant to diaminopyrimidine (drug class of Trimethoprim), two Aminoglycosides genes *gidB* and *S12p* are resistant to streptomycin, two cycloserine resistant genes (*Alr* and *Ddl*) and two fluoroquinolones and quinolones resistant genes (*gyrA* and *gyrB*) were identified in the genome sequences of all three studied strains. Other notable antibiotic-resistant genes identified in the genome sequences of the studied strains were *rho, EF-G, EF-Tu, MurA, Iso-tRNA, folP*, *VanG* and *ampS.* These genes were found to be resistant against bicyclomycin, fusidic acid, elfamycins, fosfomycin, mupirocin, sulfonamides, and vancomycin and beta-lactamases antibiotics, respectively.

### Secondary metabolites

Secondary metabolites often are considered potent sources of virulence factors for a pathogen. Although no potential gene cluster responsible for the biosynthesis of microbial metabolites were found in the strain BFF1B1 by using the antiSMASH software, two gene cluster were identified in the BFFF11, including NRPS (Nonribosomal peptides synthetases) and bacteriocin. Furthermore, a putative bacteriocin gene cluster linked to the potentially interested metabolites was found in the genome sequence of BFPS6.

### Bacteriophages

Prophages are bacteriophage mediated mobile genetic elements generally transferred by the transduction process and enable bacteria to obtain antibiotic-resistance or virulence genes. Bacteriophage helps bacteria to become more pathogenic and adapt to a new environment. Bacteriophage related antibiotic-resistance factors were demonstrated by using a phage search tool PHASTER and summarized in Supplementary Table S4. Genomes of the studied *Enterococcus* strains harbored one incomplete phage region with length 14.6 Kb and GC content ranging from 38.68 to 38.76%. Although one incomplete prophage was identified from genome sequences of BFFF11 and BFF1B1, BFPS6 conserved two incomplete regions. Interestingly, all strains conserved equal length (14.6 kb) of the incomplete region and hit a similar number (15) of phage proteins. The genome of strains BFFF11 and BFPS6 contained one putative intact phage with sizes 40.3 and 37.4 Kb, respectively.

### Insertion sequences

Insertion sequences (IS) are small mobile genetic elements that are widely distributed in the bacterial genome. Several groups of IS are found in the genome sequence of bacteria. In the present study, 25 distinct families of insertion elements/ transposes insertions were identified in the genome of *E. faecalis* strains (Supplementary Table S3). Among these, 14 families (*IS3, IS4, IS5, IS6, IS200/IS605, IS256, IS481, IS607, IS630, IS701, IS1595, ISL3, ISKra4* and *ISNCY*) of transposases were common to the three strains, 3 families (*IS30, IS66, IS91*) were in BFF1B1 and BFFF11, two were (*IS982* and *IS1182*) in the BFFF11 and BFPS6 and rest 6 families (*IS1, IS110, IS1380, IS1634, ISAs1* and *Tn3*) were found only in the strain BFFF11. In the case of a frequency distribution of IS families, BFPS6 harbored the relatively highest number of IS (N = 115), followed by BFFF11 (N = 85) and BFF1B1 (N = 46). IS family IS200/IS605 were identified as a higher frequency in the strains BFFF11 and BFF1B1 which were 14 and 12, respectively. Significantly higher frequency of *IS6* (n = 37) families were identified in BFPS6, followed by *IS3* (n = 21) and IS200/IS605 (n = 16). The distribution of the IS families and their members were summarized in Supplementary Table S5.

### Phylogenetic tree

**Figure 4:**
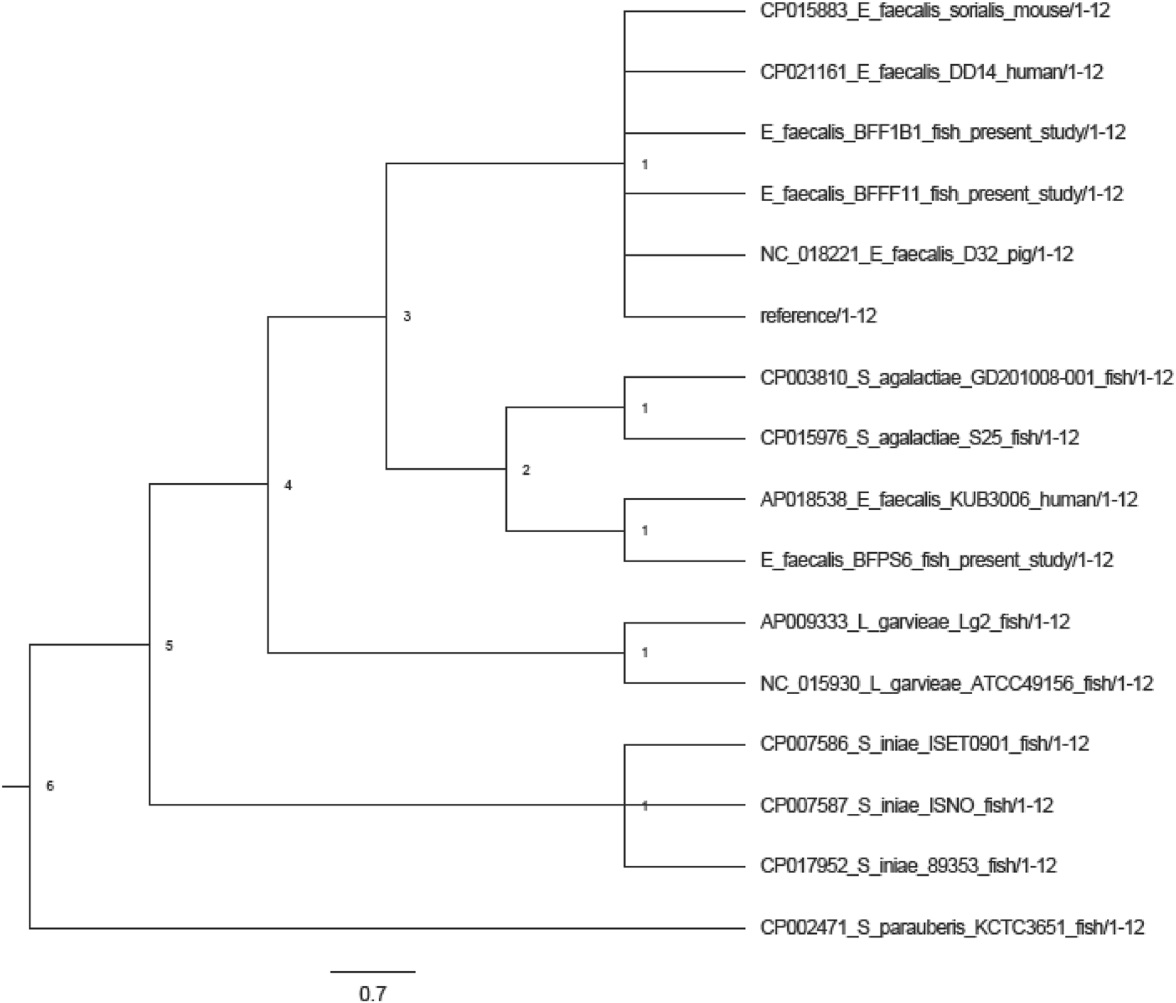
The SNP based phylogenetic tree was obtained from CSIphylogeny v1.4 by using reference genome of *E. faecalis* strain V583 (Accession No AE016830). The dendogram was modified by Fig.Tree v1.3.

A phylogenetic tree was constructed based on the single nucleotides polymorphisms (SNPs) analysis. To understand the degree of relatedness with other pathogenic *E. faecalis*, the whole genome sequence of human, mouse and pig were extracted from NCBI database and used for the analysis of the phylogenetic tree. As *E. faecalis* caused streptococcosis like infection in fish, fish pathogenic *Streptococcus* spp. from NCBI were also included in this analysis. In the phylogenetic tree, two distinct clades were formed among the three strains of the fish pathogenic *E. faecalis*. Tilapia originated strains BFF1B1 and BFFF11 formed a common cluster with the reference strain whereas BFPS6 formed a separate branch closely related to a human originated strain (Fig. 4). Although common disease symptoms were found, fish originated *Streptococcus* spp. showed a distinct out-group from the *E. faecalis* pathogen in the phylogenetic study. The average percentage of reference genome covered by all strains was 0.0292%, whereas, in the present study, strains BFF1B1, BFFF11 and BFPS6 covered 78.675, 83.747 and 80.419%, respectively.

## Discussion

Streptococcosis is caused by a complex group of bacteria where *Enterococcus faecalis* is one of the contributing pathogens of the disease. The present study highlighted the genomic features, the virulence associated genes, antibiotic-resistance genes and transfer of genetic elements of three strains of *E. faecalis* associated with streptococcosis in tilapia and silver barb fishes. The genome size of the *E. faecalis* strains obtained in this study lies between 2.8 and 3.06 Mb that are similar to the size of *E. faecalis* reported by the other studies^17,18,20^.

Virulence factors are the degree of pathogenicity of an organism that is responsible for establishing a disease in the host by combating immunity. A large group of genes conferring virulence factors were found in the genome of *E. faecalis* strains of the present study. Enterococci conserved genes responsible for biofilm formation have a major role in the pathogenicity and infection as they contribute to virulence and antimicrobial resistance activity^21^. Mature biofilms contribute to survival against antimicrobial substances at 10–1000-fold greater concentrations compared to the required dose to inhibit planktonic bacteria^22^. A number of genetic factors are linked to the production of biofilm in enterococci pathogenesis. Several genes were identified from the present study responsible for biofilm formation including aggregation substance (*agg*), endocarditis and biofilm-associated pili genes (*ebpA, ebpB, ebpC*), collagen adhesion precursor (*ace*), sortase (*SrtA*). Similarly, these biofilm conferring genes were isolated from *E. faecalis* strains isolated from human, macaques and bovine feces^20,23^. Biofilm producing genes *bopD* and serine protease *sprE* were isolated from BFPS6 in the current study. Similar genes were also recorded in the *Enterococcus faecalis* strain OG1^24,25^. Transcriptional regulators encoded gene *Psr* identified in the genome of *E. hirae*^26^*,* play key roles in cell envelope homeostasis, stress tolerance, biofilm formation and modulating the expression of genes^27^. Likewise, this gene was also found in the genome sequences of *E. faecalis* in the current study.

Hyaluronidase enzymes are the extracellular hyaluronidase that can degrade a major body matrix fluid hyaluronic acid. Two genes encoding for extracellular hyaluronidase *hylA* and *hylB* were found in the genome sequence of BFFF11 and BFPS6 while the latter gene was conserved in the strain BFF1B1. Extracellular hyaluronidase encoded genes were reported from the whole genome sequence of *E. faecalis* strains isolated from humans^18^, macaque^20^ and fish pathogenic *Listeria* sp.^28^, *Streptococcus* sp.^29^ and *Aeromonas* sp.^30^.

*Enterococcus faecalis* strains encoded three *agr*-like genes (*fsrA, fsrB and fsrC*) which are associated with the quorum-sensing mechanism and can control the expression of two virulence genes comprising gelatinase (*gelE*) and serine protease (*sprE*)^31^. Among the three quorum-sensing system signal molecule precursors, *fsrB* influences the expression of several genes all over the growth phases of bacteria^24^. These interlinked genes (*fsrA, fsrB, fsrC, gelE and sprE*) were also identified in all strains in the present study.

Several virulence genes involved in DNA and protein synthesis were identified from the present study strains. Two genes *recQ1* and *phrB* that are encoded for enzymes and could be responsible for DNA damage control and repair^32^. It was found that the loss of these genes in the pathogens play a role in the killing of a host^33^ and have sensitivity to the macrophages for oxidative burst^34^. Furthermore, transcriptional repressor coded genes *scrB*-1 and *scrR*-1 were also found in the present study strains which involved in virulence and stress response of bacteria. The above mentioned four virulence genes were also identified from strains of *E. faecalis* isolated from other sources^32,35,36^.

Many pathogenic bacteria conserve capsular polysaccharide encoding genes to evade phagocytosis and contribute a significant role for pathogenesis through immune evasion. Capsule producing genes were also harbored in all strains of the current study. Similar results were reported from different strains of *E. faecalis*^37^. Genes encoded for capsular polysaccharides are also found in a number of fish pathogenic bacteria including *cpsA* from *S. agalactiae*^38^*, cpsA, cpsB, cpsC, cpsD, cpsE* and *cpsF* in the *cps* loci of *S. parauberi*^39^.

Enterococci are reported as resistant to a wide group of antimicrobial compounds. In the present study, multiple genes conferring to tetracycline resistance found in the strain BFPS6 revealed that the strain is highly resistant to the tetracycline group of antibiotics compared to the strains BFF1B1 and BFFF11. Multiple genes responsible for antimicrobial resistance found in the genome sequences of *E. faecalis* isolated from infected humans were recorded as resistant to four dominant groups of antibiotics including erythromycin, clindamycin, tetracycline and quinupristin/ dalfopristin^18^. Woods, et al.^20^ identified multidrug resistant *E. faecalis* including macrolide resistant genes *lsa(A)* and *erm(B)*, tetracycline resistant genes, *tet(M)*, *tet(S)*, and *tet(L)*, chloramphenicol resistance gene *cat,* and trimethoprim-sulfamethoxazole resistant gene *dfrG* from a wide range of macaques (*Macaca mulatta*) samples. Multiple antibiotic-resistant genes were also reported from fish pathogenic *Aeromonas* sp. Sulfonamide-trimethoprim-resistant gene (*aadA5)* and trimethoprim resistantgenes (*dfrA* and *dfrB)* were identified from fish pathogen *Aeromonas* sp.^40^*. De, *et al*.*^41^ reported macrolide resistance gene *mph*(*A*), tetracycline resistant genes *tet(A)* and *tet(E)* and Beta-lactam resistant gene *blaOXA-12* from *A. veronii* strain Ae52 isolated from goldfish in Japan.

Bacteria have the ability to transfer their antimicrobial resistance and virulence genes to other bacteria through the gene transfer mechanisms (i.e. transfer of plasmids or transposons elements and presence of bacteriophage). These may lead to complications for infection control by reducing the effectiveness of antibiotic treatment. In the case of bacteriophage analysis with PHASTER, no antibiotic-resistant genes were observed in the prophage regions among all three strains in the current study. Therefore, it is assumed that there is a low risk of transferring AMR to other bacteria. In the present study, BFPS6 conserved more virulence factors (Numbers) indicating that BFPS6 may be more virulent compared to the two other strains of *E. faecalis*.

Enterococci cause streptococcosis like disease in fish which may also cause by *Streptococcus* sp. and *Lactococcus* sp. Phylogenetic analysis showed that the *E. faecalis* strains isolated from fish formed clusters with the strains of *E. faecalis* isolated from other sources rather than the fish pathogenic *Streptococcus* sp. and *Lactococcus* sp. Furthermore, only very few virulent and antibiotic-resistance genes showed similarities among the different genera of bacteria associated with streptococcosis in fish.

Initially, it was believed that *Streptococcus* sp. and *Lactococcus* sp. are involved in streptococcosis infection in fish. Therefore, most of the previous studies were focused on the whole genome sequence of the fish pathogenic *S. agalactiae*^29^, *S. iniae*^42^, *S. Parauberis*^39^ and *L. garvieae*^43^. Recently, *Enterococcus* was highlighted as a causative agent for streptococcosis like infection in a different region of the world. Thus, it is necessary to explore the whole genome sequence of *Enterococcus* responsible for streptococcosis in fish to identify their genomic resemblances to the other species. Primarily, a deviation was observed relative to the distribution of virulence factors among different species linked to streptococcosis in fish from different geographical areas. Phenotypically similar strains of the same or different species differ in a certain set of virulence gene clusters. Although, *Streptococcus* sp. and *Enterococcus* sp. produce similar disease symptoms in fish, very few similarities are found in their virulence genes among species as well as the genera level. One of the important findings of this study is that all three-identified strains of *Enterococcus* showed a high level of virulence, although they were isolated from two different types of fish species.

In conclusion, whole-genome sequence (WGS) analysis revealed all aspects of genomic characteristics including virulent genes, antibiotic-resistant genes and other functional genes, different categories of proteins and transposable elements. This is one of the first studies that explore the WGS of *E. faecalis* strains isolated from streptococcosis infected fish. According to the virulence genes and antibiotic-resistance genes contents, all three strains appeared to be pathogenic. All three strains possess multidrug resistant genes and were resistant to the sixteen predominantly used antibiotics in aquaculture. Tetracycline resistance genes *Tet(L), Tet(M), tet(S)* and *tet(45)* were only found in the strain BFPS6. The findings of this study show a way of quick identification of virulence factors of streptococcosis in Nile tilapia so that essential preventive measures can be taken in time.

## Methods

### Bacterial strain selection and culture

We selected three fish pathogenic strains of *E. faecalis* isolated from diseased Nile tilapia (*Oreochromis niloticus*) (BFF1B1 and BFFF11) and Thai sarpunti (*Barbonymus gonionotus)* (BFPS6) suffering from streptococcosis^5,8^. These strains were collected from three different locations of Bangladesh at different time point during summer season. The isolation and identification of bacteria were performed according to our previous work^5,8^. The strains were stored at −80 °C with 10% glycerol supplement in nutrient broth. Bacteria were routinely sub-cultured in nutrient broth (Liofilchem S.r.l., Via Scozia, Italy) and nutrient agar media (Hi Media Laboratories Pvt. Ltd., India) for 48 h.

### DNA Extraction and whole-genome sequencing

The genomic DNA was extracted from the bacteria cultured in nutrient broth using the GeneJET Genomic DNA Purification Kit (Thermo Fisher Scientific, Vilnius, Lithuania). The quality and quantity of the extracted DNA were determined by using a 0.8% agarose gel and a NanoDrop spectrophotometer (Thermo Fisher Scientific, USA). The extracted gDNA were stored at −80 °C until whole-genome sequencing. The genome sequence was performed using a MiSeq sequencer (Illumina, Inc.) according to Akter et al.^19^.

### Assembly and annotation of raw reads

Initially, the bacteria were identified by using the Bacterial Analysis Pipeline version 1.0.4^44^. The de novo assembly of the high-quality reads was performed into draft genomes with SPAdes version 3.9.0^45^. The draft genomes of the bacteria were annotated by using Prokka software tools with version 1.11.0^46^ and PATRIC, a RASTtk-enabled Genome Annotation Service^47^. Functional annotation of predicted protein was evaluated using BLASTKOALA of Kyoto Encyclopedia of Genes and Genomes (KEGG) (https://www.kegg.jp/blastkoala/)^48^. The predicted genes by KEGG functional annotation were constructed with ggplot2 using R statistical package version 3.2.3.

### Genomic comparison

The shared and unique genes among the three strains of *E. faecalis* were analyzed using an online database called RAST (Rapid Annotation using Subsystem Technology (http://rast.nmpdr.org/rast.cgi)^49^. For the comparative study among the unique genes of these pathogens were obtained from the annotated genome in SEED Viewer (http://rast.nmpdr.org/seedviewer.cgi)^49^. Plasmid replicons for the genome sequences were studied by using PlasmidFinder (https://cge.cbs.dtu.dk/services/PlasmidFinder/) with the setting of the threshold for a minimum 95% identity over 60% coverage of length^50^. Secondary metabolite biosynthetic gene clusters were analyzed by using the Antibiotics and Secondary Metabolite Analysis Shell V 5.1.2 (antiSMASH, https://antismash.secondarymetabolites.org/#!/start)^51^.

Identification of prophage associated Gene clusters in the genome sequences of the strains were identified using PHASTER server (http://phaster.ca/) ^52^. Three scenarios for the completeness of the predicted phage-associated regions were defined according to how many genes/proteins of a known phage the region contained: intact (≥ 90%), questionable (90–60%), and incomplete (≤ 60%). The comparative genomic feature was visualized using BLAST Ring Image Generator (BRIG) version 0.95 with *E. faecalis* V583 (Accession No AE016830) as reference^53^. Further, the ISfinder database (http://www-is.biotoul. fr/is.html) were used to identify the insertion sequences (IS) of the study bacteria^54^.

### Assembly and identification of virulence gene

The virulence genes were identified in the de novo assembled contigs of the *E. faecalis* strains using the web-service VirulenceFinder 2.0 (https://cge.cbs.dtu.dk/services/VirulenceFinder/) and VFanalyzer of virulence factor database (VFDB, http://www.mgc.ac.cn/VFs/)^55^ and Pathosystems Resource Integration Center (PATRIC) 3.4.2^56^ and more than 90% subject coverage and query coverage were used to select the virulence genes. For the Virulence Finder identification thresholds were set 90% over a minimum identity length of 60%. The heat maps were constructed based on the presence and absence of virulence genes in the respective strains and according to the database used to identify the genes by R software.

### Identification of antibiotic-resistance gene

Antibiotic-resistance genes in whole-genome sequence data were identified by using an online-based database named ResFinder 3.1 (https://cge.cbs.dtu.dk/services/ResFinder/)^57^, Antibiotic-resistance Gene-ANNOTation V6 (ARG-ANNOT, https://ifr48.timone.univ-mrs.fr/blast/arg-annot_v6.html)^58^, Comprehensive Antibiotic-resistance Database CARD (CARD, https://card.mcmaster.ca/analyze/rgi)^59^ and PATRIC 3.4.2^56^. The acquired antimicrobial resistance genes of all of the strains were identified using 90% nucleotide identity for ResFinder.

### Analysis of Phylogenetic tree

A Phylogenetic tree was constructed based on single nucleotide polymorphism (SNP) analysis using the web-tool CSI Phylogeny v1.2 (https://cge.cbs.dtu.dk/services/CSIPhylogeny/) ^60^. The phylogenetic tree was modified in FigTree v1.4.2 (http://tree.bio.ed.ac.uk/software/figtree/). As an output of the analysis, a matrix including the counts of nucleotides difference for all sequences was obtained. A reference sequence alignment (Accession No. AE016830) was used to generate a phylogenetic tree from the whole-genome sequence data of the strains of the present study including some other sequences obtained from the NCBI database. The SNP tree server allows setting few input parameters to filter SNPs and the default values were used according to Kaas et al.^60^.

## Supplementary Information

Supplementary Information 1.

Supplementary Information 2.

Supplementary Information 3.

Supplementary Information 4.

Supplementary Information 5.

Supplementary Information 6.

## Data Availability

The whole-genome sequence data of BFFF11, BFF1B1 and BFPS6 have been deposited in the NCBI Gene Bank under accession numbers CP045918, CP046022 and JADBGH010000000, respectively.
