# Supplementary material for: Virulence and antibiotic-resistance genes in Enterococcus faecalis associated with streptococcosis disease in fish
Source: Sci Rep. 2023 Jan 27;13:1551. doi: 10.1038/s41598-022-25968-8 (PMC9883459; doi:10.1038/s41598-022-25968-8)
Supplement: Supplementary file 1 — Supplementary Information 1. [file 41598_2022_25968_MOESM1_ESM.docx]

| Test Type | Tests | | Characteristics | | |
| --- | --- | --- | --- | --- | --- |
|  |  | | BF1B1 | BFFF11 | BFPS6 |
| Colony Characteristics | Size | | M | M | M |
|  | Type | | R | R | R |
|  | Color | | Dark red | Dark red | Dark red |
|  | Shape | | C | C | C |
| Morphological Characteristics | Shape | | Cocci | Cocci | Cocci |
| Physiological Characteristic | Motility | | - | - | - |
|  | Growth in 6.5% NaCl | | + | + | + |
|  | Growth at 10 °C | | + | + | + |
|  | Growth at 45 °C | | + | + | + |
|  | Growth in 40% bile salt | | + | + | + |
|  | Growth in 0.1% methylene blue milk at pH 9.6 | | + | + | + |
| Biochemical Characteristic | Gram’s Staining | | + | + | + |
|  | Gram’s test | | - | - | - |
|  | Catalase | | - | - | - |
|  | Oxidase | | - | - | - |
|  | Oxidative-Fermentative | | F | F | F |
|  | Methyl Red | | + | + | + |
|  | Voges-Proskauer | | + | + | + |
|  | Indole | | + | + | - |
|  | Hemolyis | | β | β | β |
|  | Urease | | - | - | - |
| Hydrolysis of |  | Esculin | + | + | + |
|  |  | L-arginine | + | + | + |
| Acid Production from |  | Arabinose | - | - | - |
|  |  | Fructose | - | - | - |
|  |  | Glucose | + | + | + |
|  |  | Inositol | - | - | - |
|  |  | Inulin | - | - | - |
|  |  | Lactose | + | + | + |
|  |  | Mannose | + | + | + |
|  |  | Mannitol | + | + | + |
|  |  | Sorbitol | + | + | + |
|  |  | Raffinose | - | - | - |
|  |  | Rhamnose | + | + | + |
|  |  | Xylose | - | - | - |
| Pathogenicity  (Mortality %) |  |  | 94 | 84 | 82 |

**Supplementary Table 1**

Colony, morphological, physiological, biochemical characteristics and pathogenicity of three strains of *Enterococcus faecalis*.

M, medium; R, round; C, convex; F, Fermentative; +, positive; −, negative.
