# Supplementary material for: Virulence and antibiotic-resistance genes in Enterococcus faecalis associated with streptococcosis disease in fish
Source: Sci Rep. 2023 Jan 27;13:1551. doi: 10.1038/s41598-022-25968-8 (PMC9883459; doi:10.1038/s41598-022-25968-8)
Supplement: Supplementary file 2 — Supplementary Information 2. [file 41598_2022_25968_MOESM2_ESM.docx]

**Supplementary Table 2.** Antibiogram profile of fish pathogenic *E. faecalis* strains BFFF11, BF1B1 and BFPS6

| **Inhibition zone ratio for tested antibiotics** | | | | | | | | | | | | |
| --- | --- | --- | --- | --- | --- | --- | --- | --- | --- | --- | --- | --- |
| **Isolates** | **Amoxycillin (AMX)** | **Ampicillin (AMP)** | **Azithromycin (AZM)** | **Cefradine (CH)** | **Cefuroxime**  **(CXM)** | **Erythromycin (E)** | **Gentamicin (GEN)** | **Levofloxacin (LE)** | **Nitrofurantoin (NIT)** | **Penicillin (P)** | **Vancomycin (VA)** |  |
| BF1B1 | R | R | 2.3 ± 0.0 | 2.2 ± 0.1 | R | R | 3.3 ± 0.1 | 3.4 ± 0.1 | 3.1 ± 0.1 | R | 2.2 ± 0.1 |  |
| BFFF11 | R | R | 2.2 ± 0.1 | 2.2 ± 0.1 | R | R | 3.3 ± 0.1 | 3.5 ± 0.1 | 3.2 ± 0.0 | R | 2.30±0.1 |  |
| BFPS6 | R | R | 2.38±0.2 | R | R | R | 2.83±0.2 | 2.61±0.1 | 3.61±0.2 | R | 3.83±0.0 |  |

Amoxicillin (30 µg disk−1), Ampicillin (25 µg disk−1), Azithromycin (30 µg disk−1), Cefradine (25 µg disk−1), Cefuroxime (30 µg disk−1), Erythromycin (15 µg disk−1), Gentamicin (10 µg disk−1), Levofloxacin (5 µg disk−1), Nitrofurantoin (30 µg disk−1), Penicillin (10 µg disk−1), Vancomycin (30 µg disk−1). R, Resistant. Disk diameter was 6.0 mm. Data are presented as Mean ± SE (n = 3).
