# Supplementary material for: Virulence and antibiotic-resistance genes in Enterococcus faecalis associated with streptococcosis disease in fish
Source: Sci Rep. 2023 Jan 27;13:1551. doi: 10.1038/s41598-022-25968-8 (PMC9883459; doi:10.1038/s41598-022-25968-8)
Supplement: Supplementary file 4 — Supplementary Information 4. [file 41598_2022_25968_MOESM4_ESM.docx]

Supplementary table 2. Summary of the prophage regions found in the bacteria *E. faecali*strains BFF1B1, BFFF11 and BFPS6 identified by PHASTER server (<http://phaster.ca/>)

| Bacterial strains of *E. faecalis* | Region | Region Length  (Kb) | GC % | Protein | Phage Hit Proteins | Hypothetical Proteins | Phage Species | Position |
| --- | --- | --- | --- | --- | --- | --- | --- | --- |
| BFF1B1 | incomplete | 14.6 | 38.76 | 18 | 15 | 3 | 10 | 198438-213067 |
| BFFF11 | intact | 40.3 | 33.65 | 54 | 39 | 15 | 25 | 169735-210079 |
|  | incomplete | 14.6 | 38.70 | 17 | 15 | 2 | 10 | 1568594-1583222 |
| BFPS6 | intact | 37.4 | 35.10 | 54 | 40 | 14 | 25 | 96929-134408 |
|  | incomplete | 14.6 | 38.68 | 17 | 15 | 2 | 10 | 1175731-1190355 |
|  | incomplete | 17.2 | 34.95 | 16 | 8 | 8 | 7 | 2119455-2136700 |
