# Supplementary material for: Virulence and antibiotic-resistance genes in Enterococcus faecalis associated with streptococcosis disease in fish
Source: Sci Rep. 2023 Jan 27;13:1551. doi: 10.1038/s41598-022-25968-8 (PMC9883459; doi:10.1038/s41598-022-25968-8)
Supplement: Supplementary file 6 — Supplementary Information 6. [file 41598_2022_25968_MOESM6_ESM.docx]

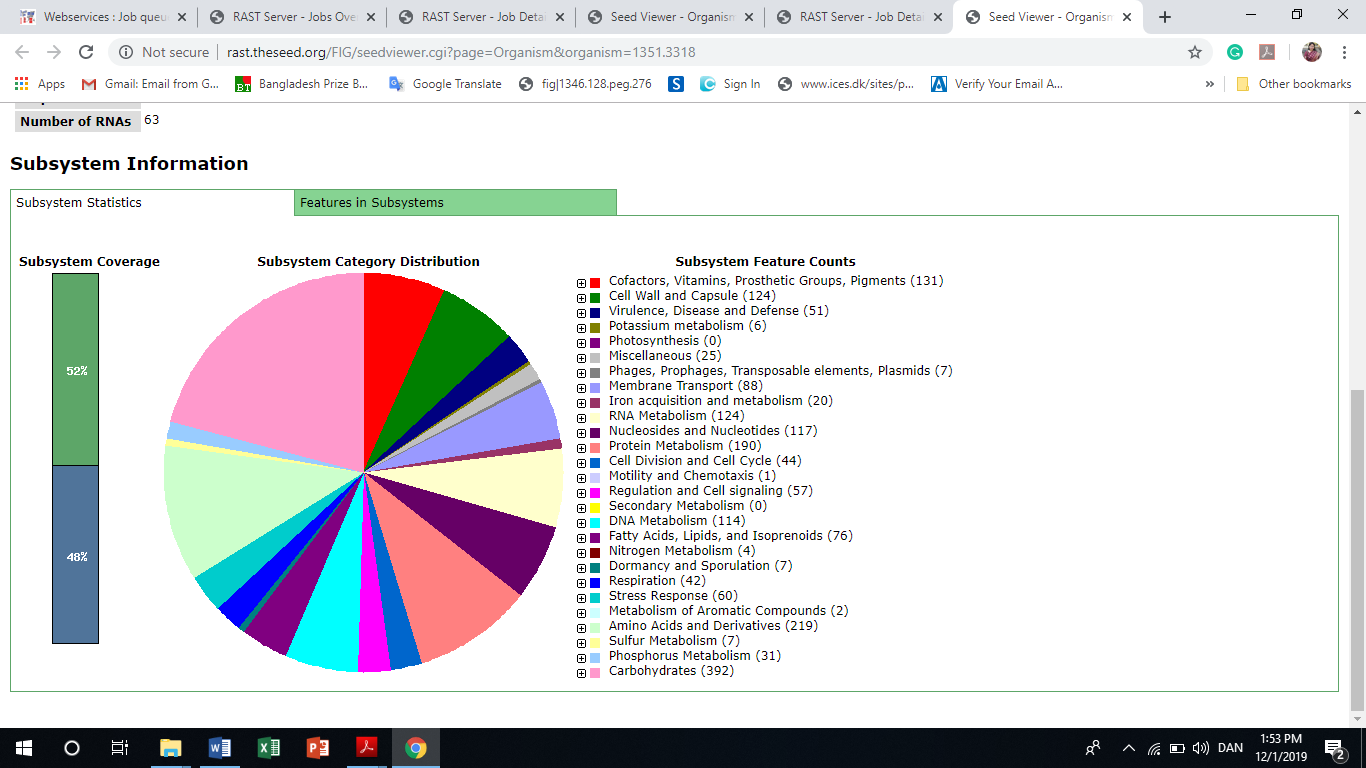


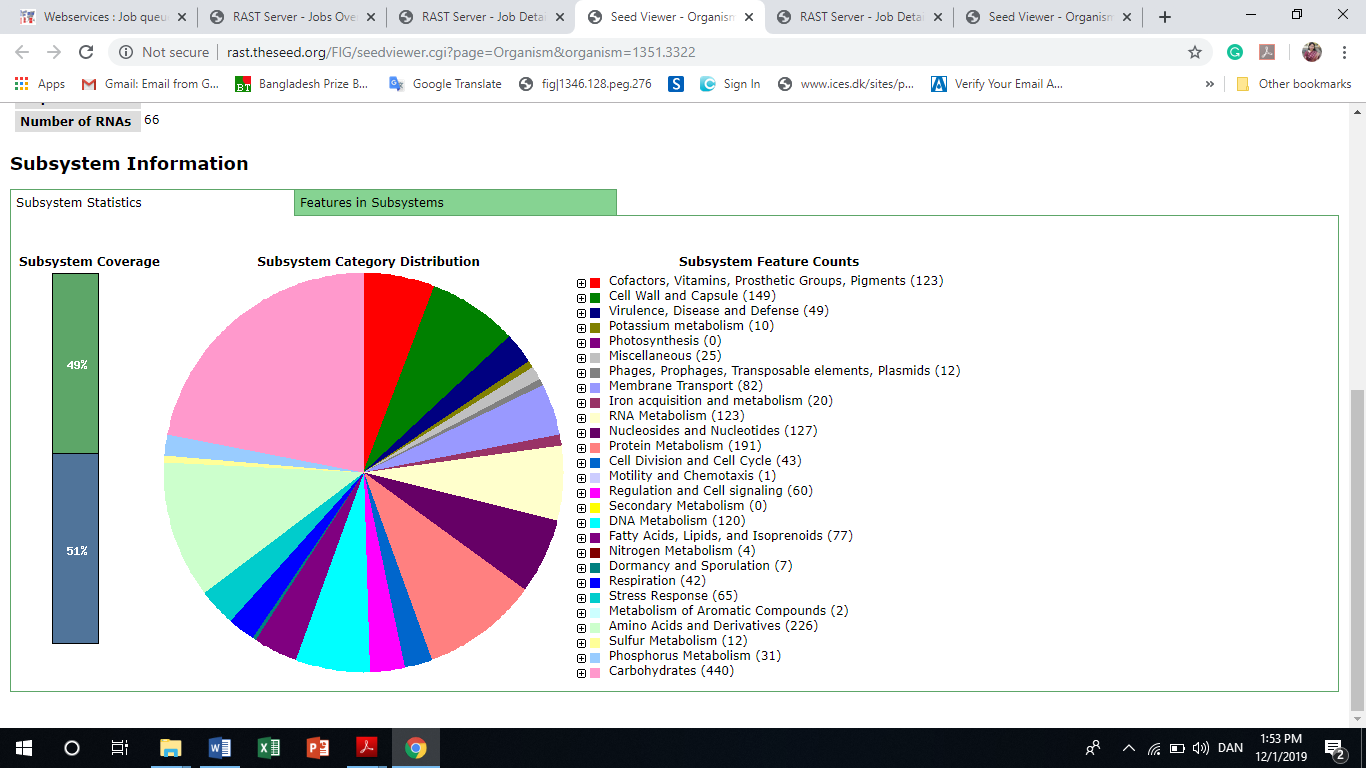


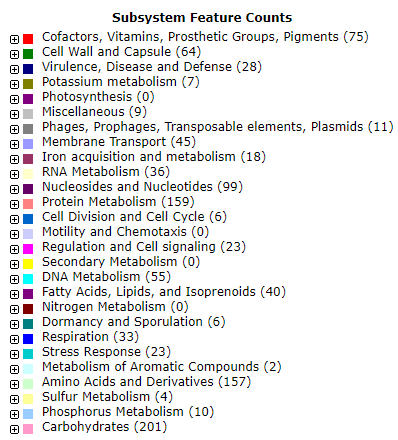

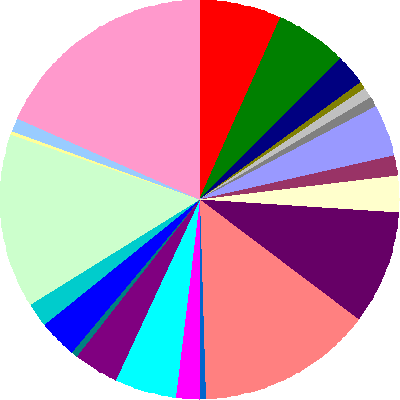

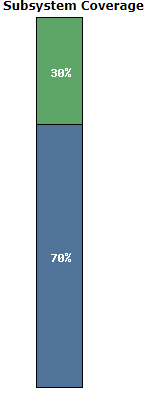


Supplementary Fig.1. Seed viewer obtained from RAST (Rapid Annotation using Subsystem Technology) (Overbeek *et al.*, 2014) analysis of the whole genome sequence of *E. faecalis* strains a) BFF1B1 b) BFFF11 and c) BFPS6.
